# Mechatronic Pole System for Monitoring the Correctness of Nordic Walking

**DOI:** 10.3390/s23208436

**Published:** 2023-10-13

**Authors:** Sławomir Wudarczyk, Marek Woźniewski, Agnieszka Szpala, Sławomir Winiarski, Jacek Bałchanowski

**Affiliations:** 1 Department of Fundamentals of Machine Design and Mechatronics Systems, Wroclaw University of Science and Technology, Łukasiewicza 7/9 Street, 50-371 Wrocław, Poland; jacek.balchanowski@pwr.edu.pl; 2 Department of Physiotherapy in Surgical Medicine and Oncology, Wroclaw University of Health and Sport Sciences, Paderewskiego 35 Avenue, 51-612 Wrocław, Poland; marek.wozniewski@awf.wroc.pl; 3 Department of Biomechanics, Wroclaw University of Health and Sport Sciences, Mickiewicza 58 Street, 51-684 Wrocław, Poland; agnieszka.szpala@awf.wroc.pl (A.S.); slawomir.winiarski@awf.wroc.pl (S.W.)

**Keywords:** gait analysis, Nordic walking, mechatronic system, sensors, foot reaction forces, NW pole reaction forces

## Abstract

Marching with Nordic walking (NW) poles is a common form of physical activity. It is recommended in the treatment and rehabilitation of many diseases. NW’s wide range of applications in rehabilitation and its effectiveness are limited by the need for experienced physiotherapists to supervise patients during the training. A prerequisite for good rehabilitation results is correctly using the poles during walking. Essential parameters of NW include the angle of inclination of the pole, the force of the pole on the ground, and proper coordination of performed movements. The purpose of this paper is to present the design and operating principle of a mechatronic NW pole system for measuring and recording the gait parameters. The subject of the work was the assessment of the usefulness of the mechatronic NW pole system for phases identified during marching. The study was conducted in field conditions. The study’s main objective was to compare the obtained results from the developed system with those of a commercial system for measuring foot pressure distributions on the ground. The paper also presents sample results measuring walkers’ gait with NW poles in the field and the resulting gait phase analysis.

## 1. Introduction

Nordic walking (NW) is a type of physical activity that uses special poles designed for this form of movement. The great advantage of NW is that it can be practiced virtually by anyone. In recent years, NW has gained popularity and been taken up by people of various ages and different individual fitness levels. In the literature, NW is most often compared (in laboratory or field conditions) to free walking and jogging [1,2,3,4].

NW engages practically the entire human musculoskeletal system. Walking with poles activates approximately 90% of the muscles, whereas ordinary walking only engages 40–50%. This is a huge advantage of this activity. Generally, there are no contraindications to able-bodied people practicing this form of activity; the only exceptions are special cases where this type of exercise is not recommended by a doctor.

The NW march is also widely used in the rehabilitation of many medical conditions [5]. Research clearly indicates that walking with poles is one of the best forms of physical activity for hypertension [6]. The results of a study by researchers at the Cooper Institute Dallas, Texas, had significant implications for health. They compared NW with regular walking and showed an increase in energy expenditure and oxygen consumption of approximately 20% compared to regular walking at the same pace [7]. The increased energy expenditure of NW compared to normal walking is important in increasing physical fitness and reducing body weight faster [5]. Compared to regular walking, NW engages the muscular system, especially the upper body, to a greater extent, leading to an increased cardiorespiratory response in the absence of fatigue [8,9,10]. 

The correct NW technique consists of many elements. The aim of each is to force the desired movements of the entire body. Properly performed NW is characterized by elongation of upper limb movement. This is of great importance for people with respiratory conditions and for postmastectomy patients, whose chests are stiff and contracted due to reflexive increased muscle tension [11].

The benefits of NW are also observed among people with chronic spinal pain [12], whose movement to push off the pole probably ‘lengthens’ the spine, allowing for faster regeneration of the intervertebral discs. NW is also a recommended form of rehabilitation among people with cardiovascular disease, who, as a result of systematic walking, experience an increase in aerobic capacity and possibly an improvement in endothelial function [13,14]. 

Acquiring the skills needed for the correct NW technique indisputably results in a longer gait step. An observed effect of NW is an increase in walking distance and training intensity with subjective lack of fatigue and improvements to the body’s metabolism, meaning NW has applications in programs to combat overweight and obesity [9,15,16,17]. During NW, upper limb work improves the range of mobility in the shoulder joints in addition to increasing muscle strength [13,18]. The chest and shoulder muscles are stretched as a result of increased rotation of the shoulder girdle.

The research conducted on NW has largely been concerned with the correct marching technique. In scientific studies, all aspects of NW that are beneficial for health were obtained when using the proper technique [4]. In the scientific studies quoted above, their participants always received training under the supervision of qualified instructors, the purpose of which was to teach the proper use of NW poles. However, the availability of a series of professionally conducted training sessions is in many cases limited. Therefore, the rise in the popularity of NW is inevitably resulting in an increased risk of incorrect use of the poles by untrained people. In such cases, the movements performed have no therapeutic effect and may even intensify existing health problems. A common result among people who use a poor technique for a long time is their fixation on an incorrect movement pattern, which is later difficult to eliminate.

Equipping the NW poles with a system that gives objective feedback on the basic parameters of marching can support the individual development of correct gait technique, without the constant supervision of a coach. One of the most important elements in NW exercise is the correct movement of the poles in relation to the patient (change in orientation and striking the poles against the ground in the correct support phases and movement of the foot during gait).

Attempts to develop mechatronic systems to evaluate NW have been made in different research centers. An example is the system based on IMU and force sensors placed on the poles, which was presented in [19]. The system allows for measuring accelerations and pole forces on the ground during the march. However, the disadvantage of this system is the lack of information on lower limb movement (e.g., measurement of foot ground pressure forces). For this reason, it is not possible to assess the correctness of NW using this system. Only the movement of the poles alone can be assessed, separately from the movement of the lower limbs. Furthermore, mounting the force sensors requires cutting the pole. When embedding the sensor close to the handle, the axial force measurement is disturbed by bending the pole. Therefore, the proposed solution is practically limited to high-stiffness poles (e.g., aluminum alloys). The use of this solution for carbon poles seems unfeasible.

Further research work has involved attempting to alter the design of poles to change their stiffness and damping, to improve their dynamic characteristics. An example is research in which the modification of the pole consisted of equipping it with an integrated resistance shock absorber, which was used in a female gait study to test body balance [20]. 

This review of the literature clearly indicates that the wide range of applications of this rehabilitation method and its effectiveness are limited by the need for experienced rehabilitation professionals to supervise patients. The possibility to develop a correct NW technique without direct supervision from a physiotherapist will certainly expand availability and improve the effectiveness of rehabilitation with the NW method.

Equipping NW poles with a system that gives objective feedback on the basic parameters of NW can be useful to support the individual development of correct gait technique without the constant supervision of a coach, and such a system is presented herein.

The first part of this paper presents the construction and principle of operation of the developed mechatronic NW system. The task of this system is to support the process of learning the correct NW gait technique. The data stored in this system enable the trainer to quickly assess the mistakes made by the patient during individual NW (performed without the trainer’s presence).

One of the most important elements in NW is the correct movement of the pole in relation to the patient’s body. The NW pole should be driven into the ground at the correct angle and at the proper moment of the gait cycle. The change in the angle of inclination of the NW pole during its support phase is also an important element in the assessment of NW technique. The evaluation of NW pole technique is only possible in terms of the identified phases of the gait cycle.

This article present the results of tests of the module of the mechatronic NW pole system designed to identify the gait cycle phases. A commercial system for measuring foot pressure distributions in shoes was used to verify the correct operation of this module. Furthermore, this article presents the results of measurements to identify the gait cycle phases of walkers with NW poles in the field. An exemplary analysis of selected NW parameters in terms of the identified phases of the gait cycle is also presented.

## 2. Materials and Methods

**Figure 1:**
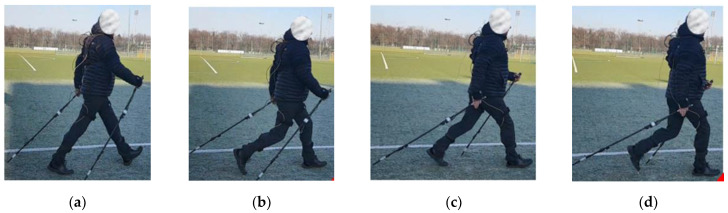
Basic phases of NW gait: (**a**) the heel of the left foot touches the ground, the angle and moment of striking the right stick; (**b**) putting the weight on the whole left foot, the beginning of the push-off phase for the right pole; (**c**) lifting the heel off and putting the weight only on the toes of the left foot, the end of the push-off phase for the right pole; (**d**) lifting the left foot off the ground, the beginning of the phase of transferring the right pole forward.

The foundation for the design of a mechatronic system for monitoring NW marching parameters is the analysis of the particular phases of the march. At its core, there are alternating arm and leg movements. As is the case in casual walking, it is possible to distinguish a support phase and a transfer phase for each leg. Similarly, it is possible to distinguish the particular phases of NW pole movement, the support phase of the pole and its transfer phase. At the same time, when designing such a system, it is important to bear in mind that it should be a system that can be commonly used. Thus, the estimated cost and ease of use are its essential features. Measuring too many parameters will generate large amounts of information, whose analysis would become a complicated process. Therefore, in this study, the selection of the most relevant parameters and quantitative and qualitative indicators that describe the NW gait technique was essential. These parameters were selected with the participation of coaches and rehabilitation therapists. The NW technique was adopted according to the guidelines of the International Nordic Walking Federation (INWA) to determine the pattern of movement. According to INWA, the following characteristic elements can be distinguished in the NW pattern, which are performed in a specific sequence or simultaneously (Figure 1):

- Placing the heel of the left leg (leading) on the ground (Figure 1a);
- Putting the weight on the whole left foot (Figure 1b);
- Lifting the heel and putting the weight on the toes of the left foot only—push-off phase (Figure 1c);
- Lifting the left foot off the ground—swing phase (Figure 1d).

The phases of leg movement are related to the phases of NW pole movement:

- The moment and angle of pushing the right pole against the ground (Figure 1a);
- Changes in the angle and force of pressure exerted by the right pole on the ground during push-off (Figure 1a–c);
- Changes in the angle of the right pole when it is moved forward (Figure 1d).

The parameters useful for the gait analysis of NW were identified through research work carried out together with the team of coaches and physiotherapists from Wroclaw University of Health and Sport Sciences [21,22]. The following left/right pole and left/right foot parameters were selected to be monitored:

- Pole inclination angle relative to the horizontal in the sagittal plane;
- Pole-to-ground contact forces;
- Feet-to-ground contact forces—measured at the heel, and at the metatarsus with the toes.

### 2.1. Construction of a Mechatronic Measuring System for NW Poles

The created mobile Mechatronic Nordic Walking Poles (MNW) system and associated computing system are designed to perform measurement, recording, and processing of selected parameters during human walking in natural conditions. During the first stage of the design process, the range of values of the measured parameters needed to be defined. In addition to the literature analysis, a number of experimental studies were carried out to determine the expected values of the measured forces and kinematic parameters. These studies were carried out using commercial measurement systems as well as a research stand developed by the authors’ team [23]. With knowledge of the measured parameters’ value ranges, it was possible to initiate the process of selecting appropriate sensors. The next step was to test selected groups of sensors for each measured physical quantity. The testing of the selected sensory elements showed that they exhibited sufficient dynamics and measurement ranges. The next step was to develop a measurement system to acquire and process the collected data.

In the developed system, sensors are placed in the poles and shoes, and the data obtained from them allow gait accuracy to be assessed. The measuring system is equipped with the necessary data recorders as well as user interfaces so that the acquired information can be easily analyzed. The system is also equipped with a wireless communication module, by which the measured gait parameters of the Nordic walker can be transmitted to an external computer.

**Figure 2:**
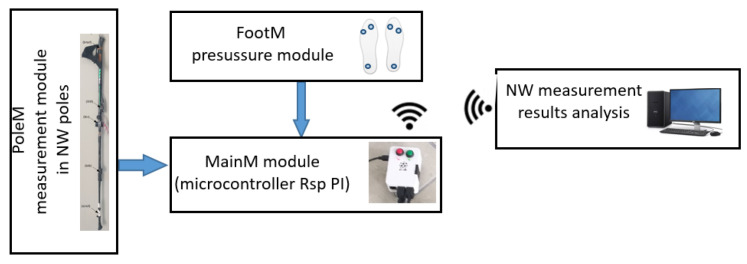
Block diagram of the mechatronic NW pole system.

Figure 2 shows a block diagram of the MNW system. The developed system includes the following modules:

- PoleM—mounted on the poles and measuring the inclination angles of the poles *Lroll/Rroll* (measured in the sagittal plane) with respect to the horizontal plane, and the *Lforce*/*Rforce* pressure of the poles on the ground (measured along the axis of the pole).
- FootM—fitted in shoe insoles and designed to measure foot contact forces on the ground: *Lfoot3*/*Rfoot3* measured in the heel; *Lfoot1/Rfoot1* and *Lfoot2/Rfoot2* measured in the metatarsal with the toes.
- MainM—the main module located in the backpack (pocket), whose task is to acquire, record, and wirelessly transmit the obtained measurement data from the PoleM and FootM modules.

**Figure 3:**
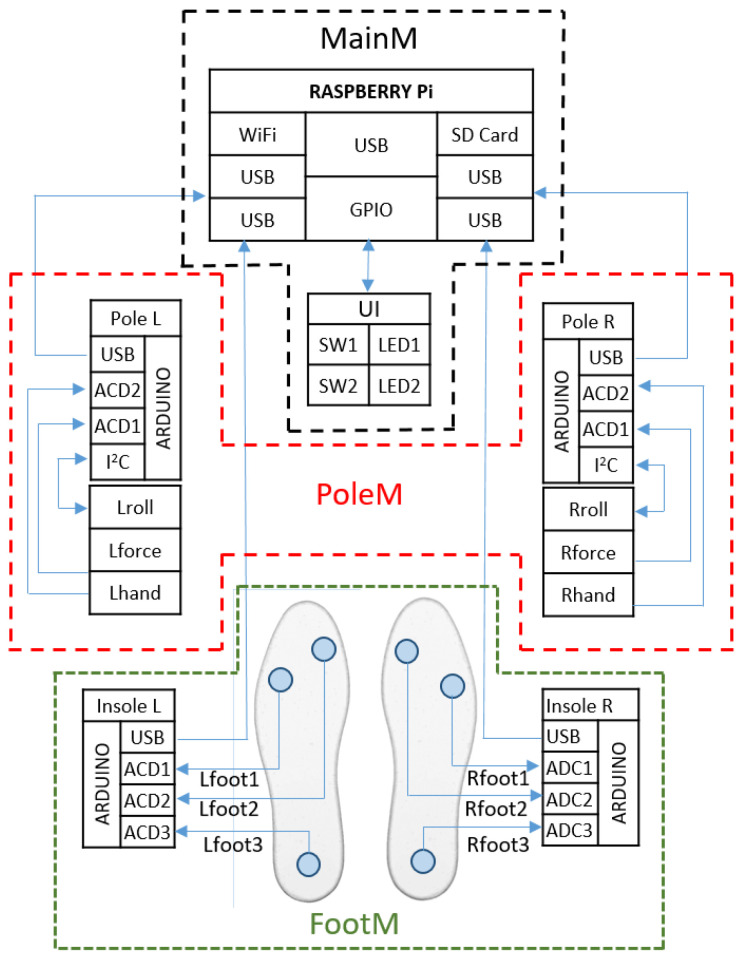
Scheme of MNW monitoring system.

Figure 3 presents a functional diagram of the mobile NW mechatronic pole system (MNW).

The PoleM module (Figure 3) was designed and manufactured at the Wrocław University of Science and Technology [21,22,23]. It consists of two identical measurement modules mounted on the left (PoleL) and right (PoleR) NW pole. To measure the angle of inclination of the pole from the horizontal, measured in the vertical (sagittal) plane, an IMU MPU9250 module is used. It is a 9-axis MotionTracking device with a 3-axis gyroscope, 3-axis accelerometer, 3-axis magnetometer, and a Digital Motion Processor™ (DMP) in a small 3 × 3 × 1 mm QFN package. MPU-9250 features three 16-bit analog-to-digital converters (ADCs) for digitizing the gyroscope outputs, three 16-bit ADCs for digitizing the accelerometer outputs, and three 16-bit ADCs for digitizing the magnetometer outputs. Communication with all registers of the device is performed using either I2C at 400 kHz or SPI at 1 MHz.

The AHRS sensor fusion algorithm, which combines gyroscope, accelerometer, and magnetometer measurements into a single measurement of orientation relative to the Earth (NWU convention) (developed by S. Madgwick [24]), was chosen as the algorithm for determining the orientation of the pole in space. This algorithm was selected after a series of tests of various algorithms as the one that performs the best to determine pole orientation during the NW gait. The results of previous field tests and those carried out on a developed test rig have shown that the measurement error of the pole inclination angle is less than 3.5° [22,23].

The contact force of the pole on the ground is measured through a UNIPULS UNCDW-500N sensor, the main technical parameters of which are listed in Table 1 [25]. It is embedded in a specially designed sleeve, which is placed at the lower end of the pole. This solution does not require interference with the structure of the pole itself and allows the use of the original pole tip (so-called spike). This way of mounting the force sensor allows for measuring the axial force of the pole on the ground—disregarding bending forces. The UNCDW-500N sensor is connected to a measuring transducer (strain gauge bridge) mounted on the NW pole, from which signals are connected to the analog input of a microprocessor. The measurement error of the axial force is less than 5.4 N [22,23].

An STM32F303 Nano microcontroller was chosen as the platform for the embedded software development. Software was developed that performs initialization of the IMU sensors, reading of values from the force sensors distributed on the pole, and transfer of data to the data aggregation system.

**Figure 4:**
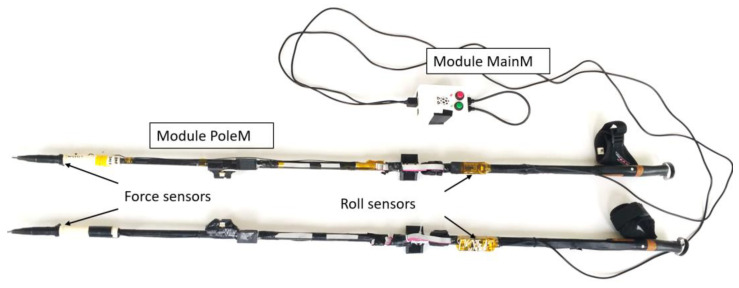
Sensor placement on the mechatronic NW pole.

Individual electronic modules were mounted on NW poles in places that allowed the desired parameters to be recorded (Figure 4) and increased the pole mass by 0.105 kg. The components were distributed in such a way that the position of the center of mass of the pole did not change.

The FootM module (Insole L, Insole R) consists of two sensor insoles and is designed to measure the distribution of foot pressure on the ground. Each insole is equipped with three FlexiForce A201 [26] pressure sensors, which are connected to an Arduino Nano. Its essential technical specifications are presented in Table 2.

The data read from each insole are sent to the MainM. The sensors are located at key points to measure the contact force of a foot on the ground. The essence of the operation of these insoles is to detect the phases of support, transfer, and distribution of foot contact force on the ground (heel, metatarsus with toes). The reason for placing two sensors in the forefoot is that the forefoot on the medial and lateral sides can be loaded highly asymmetrically (postural defects, uneven terrain, footwear type).

**Figure 5:**
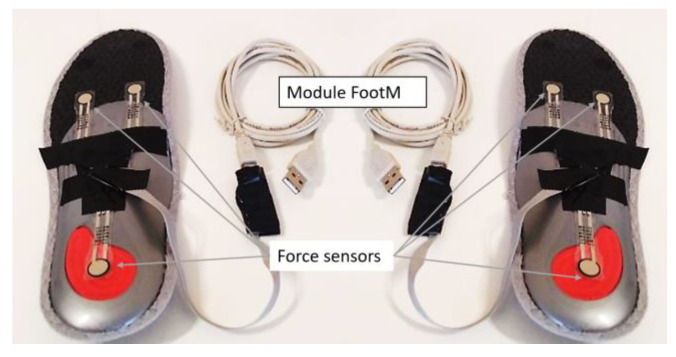
View of the foot pressure sensory insoles—FootM module.

Figure 5 shows a view of the sensory insoles for measuring foot pressure on the ground (FootM module), together with the locations of the pressure sensors.

A single-board Raspberry Pi 3B computer is used in the MainM module to store data from two sticks (PoleM module) and two measuring insoles (FootM module). The use of such a system gives great freedom in the application of different solutions: data storage, communication, and software updates. Furthermore, the use of this solution allows the implementation of a wireless connection to the entire unit via the built-in WiFi network. The MQTT protocol server can be used to send data through the wireless communication channel. The MainM module is connected via USB cables to the PoleM and FootM. This module is fitted in a special housing that allows it to be placed in the pocket or backpack of the walker. 

### 2.2. Construction of Measuring System for Verification of Sensory Insoles’ Operation

When analyzing the results of NW gait measurements, it is important to distinguish full gait cycles, which defines the fragment of gait from the heel strike of the lead leg against the ground to the repeated heel strike against the ground of the same lower limb. In the designed NW mechatronic pole system, gait cycle identification is realized by means of the FootM module. The correct operation of this measurement module under field conditions is the basis for assessing the correctness of the NW gait. The other measured parameters of NW gait are evaluated in relation to the lower limb movement cycle determined with the FootM module. For a complete analysis of the NW gait with poles, it is necessary to take into account the runs of the angles of the NW poles in the sagittal plane of the walker and the runs of the pole impact forces on the ground during a complete gait cycle.

**Figure 6:**
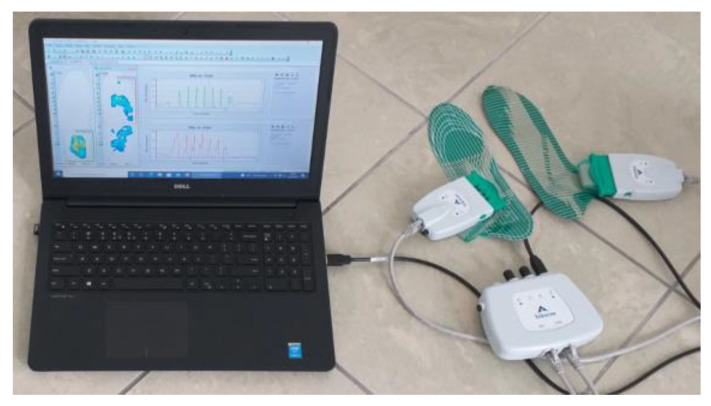
View of the components of the Tekscan F-Scan In-Shoe measurement module.

To evaluate the validity of the FootM system developed, a commercial system was used to study the distribution of foot pressure on the ground. The Tekscan F-Scan In-Shoe system, designed to measure dynamic foot pressure on the ground and analyze gait phases, was used for this purpose (Figure 6) [26]. It consists of two Tekscan Medical Sensor 3000E insoles placed in the shoe and connected via Ethernet cables to a communication module, which uses a USB connection to transfer data to a computer with F-Scan Research software (version 7.50-08). The main technical parameters of the Sensor 3000E insole are included in Table 3.

**Figure 7:**
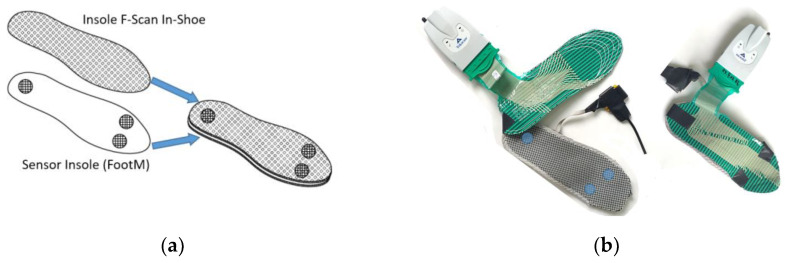
Integration of the sensory insole (FootM) and the F-Scan In-Shoe into a single measurement system ready to be placed inside shoes: (**a**) diagram, (**b**) view of the combined shoe insoles.

Before measurement, the system is individually calibrated against the weight of the examined patient. During measurement, the system captures and records contact forces and pressure distribution profiles on the surface of the insole in real time. For our tests, insoles from the F-Scan In-Shoe system and the FootM system were placed one on top of the other, appropriately taped together, and placed in the footwear (Figure 7).

**Figure 8:**
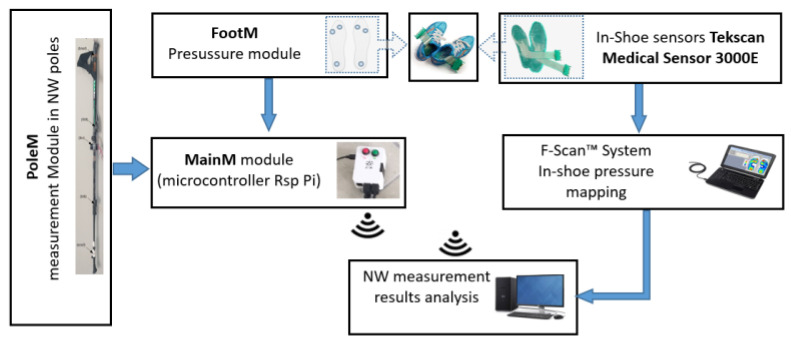
Scheme of the system for verifying the correct operation of the FootM sensory insoles.

The PoleM and F-Scan In-Shoe modules perform measurements independently on the two computer systems. These measurements are synchronized by a common force pulse marker, which is recorded by both systems simultaneously. Figure 8 shows a schematic diagram of the developed measurement system. The data acquired from both measurement systems are transmitted to a host computer, where the process of analyzing the measurement data obtained from both systems is performed. In our tests, we carried out the analysis of the acquired results offline.

### 2.3. Participants

Participants in the study were amateurs varying in gender, age (26–64 years), height (158–184 cm), and body weight (58–92 kg). None of the participants had previous NW march experience. The ethics committee approved the research procedures and all participants gave their informed written consent before participating in the research. Before the research, the participants attended a one-hour training session to learn the NW gait technique according to the guidelines of the International Nordic Walking Association (INWA). The training was carried out by a sports science professional and NW instructor with 20 years of experience. The reliability of the system was tested for ten adult persons (Table 4). 

### 2.4. Procedures of Experimental Research on NW Pole Gait

**Figure 9:**
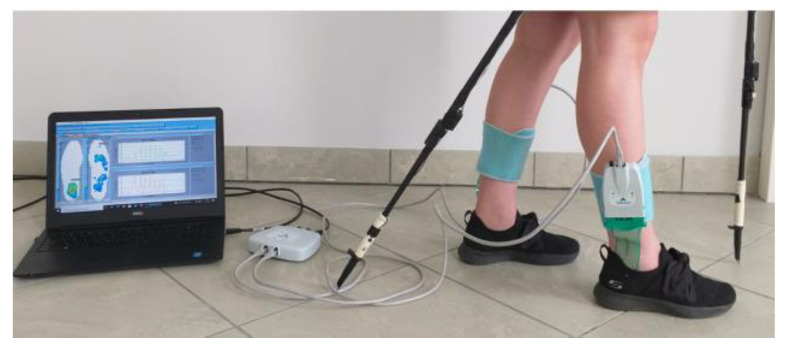
View of a person prepared for a measurement walk with NW poles equipped with elements of the PoleM and F-Scan measurement systems.

In order to verify the correct operation of the developed MNW system, real-life tests were carried out. The aim of the research was, in particular, to identify the gait cycle realized with the FootM module. The correctness of the NW gait of the walkers participating in the research was not assessed at this stage of the research. Figure 9 shows a view of a person prepared for the measurements.

**Figure 10:**
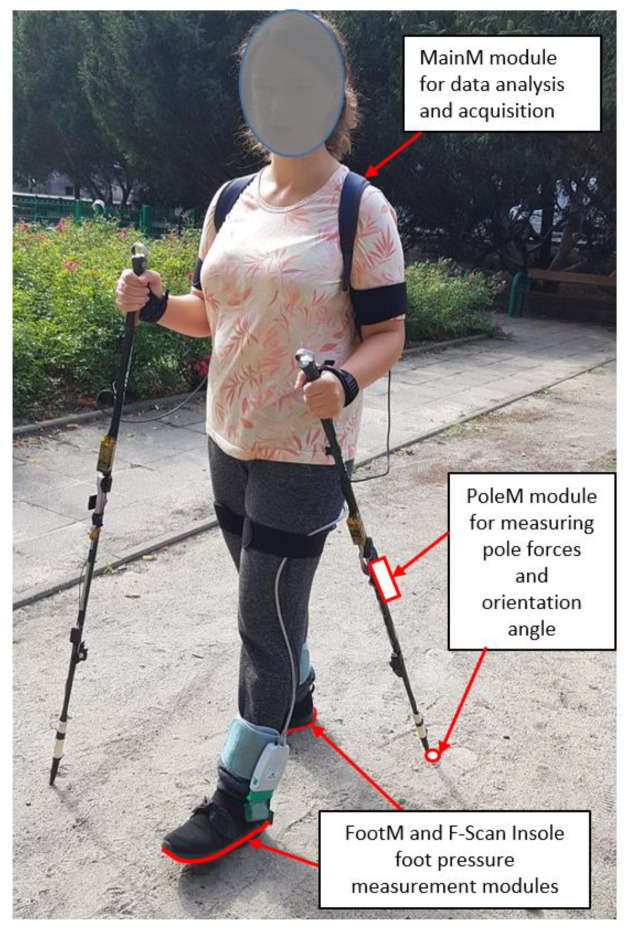
The course of the NW gait field tests.

Measurements were recorded during NW gait training in an outdoor area. The participants’ task was to walk along a park’s pathway that was 30 m long and 3 m wide. The study consisted of walking 10 straight-line sections with turns. The recording of the gait parameters was supervised by an assistant operating the FootM, PoleM, and F-Scan measurement modules (Figure 10) and the NW instructor. During each pass, the walking parameters of the NW amateurs were measured and monitored using a developed measurement system operating at 50 Hz.

**Figure 11:**
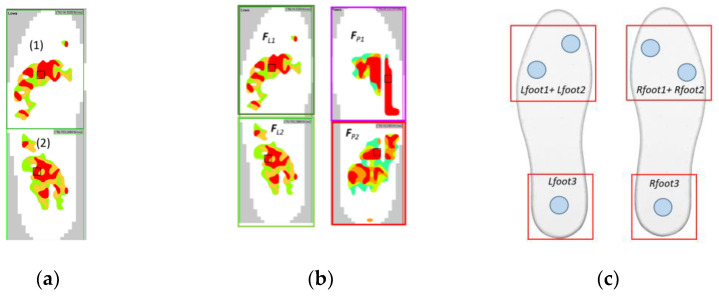
Views of (**a**) general foot pressure maps, (**b**) F-Scan subdivision of foot pressure areas into fields to determine the resultant forces *F_L1_*, *F_L2_*, *F_R1_*, and *F_R2_*, (**c**) subdivision of foot pressure areas into fields to determine the sum forces *Lfoot1 + Lfoot2*, *Rfoot1 + Rfoot2*, and *Lfoot3*, *Rfoot3* in the FootM system.

To support the analysis of the results of foot ground pressure recorded using the F-Scan In-Shoe system during walking, they were presented graphically in the form of a map with pressure levels with constant values (Figure 11a). It should be noted that from the point of view of NW gait analysis, two areas of foot pressure on the ground are most relevant: the metatarsus with toes (1) and the heel (2) (Figure 11a). Accordingly, for the analysis of the resulting foot–ground contact forces, it was decided to present the results in terms of foot interaction forces in the metatarsal with toes (*F_L1_*, *F_R1_*) and heel areas (*F_L2_*, *F_R2_* forces) (Figure 11b).

This approach allows the results obtained with the F-Scan In-Shoe system to be compared with those obtained with the MNW sensor insole (FootM) system (Figure 11c), assuming that the pressures of the metatarsal area with toes *F_L1_*, *F_R1_* are compared with the sum of the pressures from the sensors *Lfoot1 + Lfoot2* and *Rfoot1 + Rfoot2*, respectively, while the pressures of the heel area *F_L2_*, *F_R2_* are related to the pressures measured with the sensors *Lfoot3*, *Rfoot3*.

Such a comparison is obviously made in terms of gait phases and is not a quantitative comparison. The primary objective was to identify the contact forces that occur in the heel and forefoot area with the toes.

When analyzing the results of gait measurements with NW poles, it is important to distinguish full gait cycles, which defines the fragment of gait from the impact of the heel on the ground to the impact of the heel on the ground of the same lower limb again. In one gait cycle, we can distinguish between support and transfer phases. The support can be single when we lean on one limb or double when we distribute the weight between both legs. The transfer phase starts when the toe lifts off the ground and ends when the heel touches the ground again. The constructed system is universal in nature and is designed to work for people differing in anatomy. 

The developed FootM measuring system of mechatronic poles determines the moments of heel and metatarsal-with-toe impacts on the ground, with some error compared to the commercial F-Scan system.

**Figure 12:**
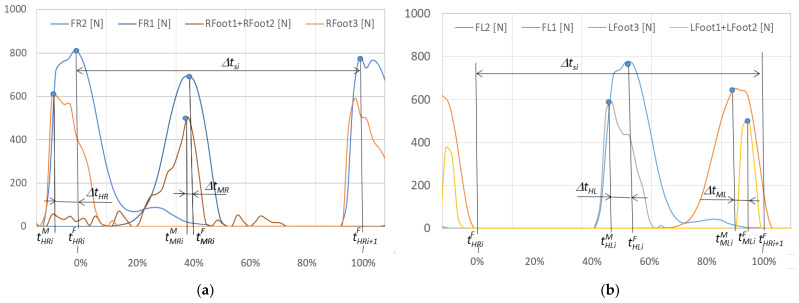
Changes in the pressure forces: (**a**) *F_R1_*, *F_R2_* and *Rfoot1* + *Rfoot2*, *Rfoot3* of the right foot, (**b**) *F_L1_*, *F_L2_* and *Lfoot1* + *Lfoot2*, *Lfoot3* of the left foot on the ground during time *t* (in % of the gait cycle) for an exemplary cycle and gait with NW.

**Figure 13:**
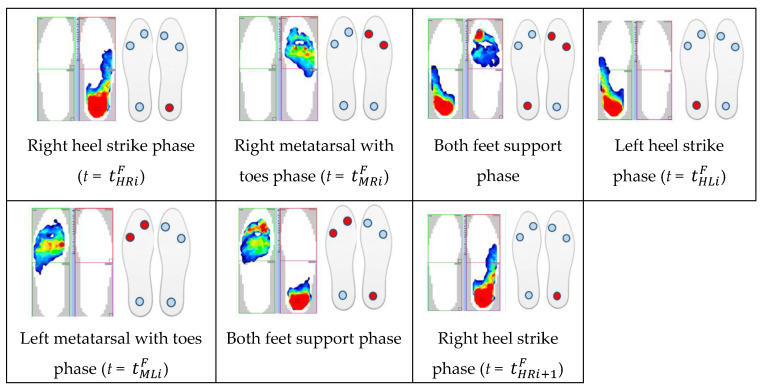
Graphical views of feet pressure maps illustrating the support and transfer phases for the selected exemplary gait cycle determined using the F-Scan and FootM systems.

In order to conduct a detailed comparative analysis of walking with NW poles, an example of a typical *i*-th gait cycle is presented. It begins with the heel of the right foot striking the ground at time $t_{HRi}^{F}$ and ends with the same heel striking the ground at time $t_{HRi+1}^{F}$. The graphs in Figure 12 show the course of changes in the pressure forces of the right and left foot on the ground measured with the F-Scan and FootM systems during one selected gait cycle. Figure 13 illustrates the foot pressure maps on the ground obtained with the F-Scan and FootM systems.

When analyzing the selected cycle, we can distinguish support phases (on the right foot, on both feet, on the left foot) and transfer phases (right and left lower limb). In the analyzed gait cycle, the support phase of the right foot begins from the moment the right heel hits the ground and lasts until the metatarsal and toes lift off the ground. Then, the phase of transfer of the right foot over the ground begins and lasts until the right heel hits the ground again, which ends the complete gait cycle (Figure 12). The phases of supporting and transfer of the left foot are defined similarly.

In order to correctly determine the support and transfer phases, it is necessary to determine the moments when the heel and metatarsal with toes strike the ground for the right and left foot in the analyzed gait cycle. The moment of strike was considered to be the time at which the pressure force reached its maximum. The moment of strike of the right heel and metatarsal with toes on the ground measured by using the F-Scan system are described, respectively, by the times $t_{HRi}^{F}$ and $t_{MRi}^{F}$. When measured with the FootM system, they are times $t_{HLi}^{M}$ and $t_{MLi}^{M}\;$(Figure 12a). The moment of impact of the left heel and the left metatarsal with the toes is determined by the times $t_{HLi}^{F}$ and $t_{MLi}^{F}$ measured by using the F-Scan system and by the times $t_{HLi}^{M}$ and $t_{MLi}^{M}$ obtained by using the FootM system (Figure 12b).

To determine the quality of the developed FootM system, basic calculations and statistical analyses should be performed comparing the obtained measurements of both systems. The duration $∆t_{si}$ of the analyzed *i-th* gait cycle (Figure 12) was assumed to be the following:

(1) $$∆t_{si}=t_{HRi+1}^{F}-t_{HRi}^{F},$$

while the average length of the gait cycle was as follows:

(2) $$∆t_{s}=1/n\sum_{i=1}^{n}∆t_{si},$$

where: 

*i*—number of the analyzed gait cycle;

*n*—quantity of measurement;

$∆t_{si}$—duration of the *i*-th gait cycle (Figure 12).

Relative errors in measuring the moment of strike of the heel and metatarsus with the toes of the right foot and the ground during the cycle, and expressed as a percentage of the length of the gait cycle (Figure 12), are described by the following formulas:

(3) $$∆t_{HXi}=\frac{t_{HXi}^{F}-t_{HXi}^{M}}{∆t_{si}}100\%,$$

(4) $$∆t_{MXi}=\frac{t_{MXi}^{F}-t_{MXi}^{M}}{∆t_{si}}100\%,$$

(5) $$∆t_{HX}=1/n\sum_{i=1}^{n}∆t_{HXi},$$

(6) $$∆t_{MX}=1/n\sum_{i=1}^{n}∆t_{MXi},$$

where: 

X = *R*, *L*—index of the analyzed foot, right—*R*/left—*L*;

$t_{HXi}^{F}$—moment of strike of the X foot heel on the ground during the *i*-th gait cycle measured with the F-Scan system;

$t_{HXi}^{M}$—moment of impact of the X foot heel on the ground during the gait cycle measured with the MFoot system;

$t_{MXi}^{F}$—moment of impact of the metatarsus with the toes of the *X* foot against the ground during the *i*-th gait cycle as measured with the F-Scan system;

$t_{MXi}^{M}$—moment of impact of the metatarsus with the toes of the *X* foot against the ground during the *i*-th gait cycle measured with the FootM system;

$∆t_{HXi}$—error in measuring the moment of heel strike of foot *X* on the ground with the FootM system relative to the measurement with the F-Scan system, expressed in % of the *i-th* gait cycle duration;

$∆t_{MXi}$—error in measuring the X metatarsal moment of impact with the Mfoot system relative to the measurement with the F-Scan system, expressed in % of the duration of the *i*-th gait cycle;

$∆t_{HX}$—mean value of the error $∆t_{HXi}$;

$∆t_{MX}$—mean value of the error $∆t_{MXi}$.

## 3. Results

**Figure 14:**
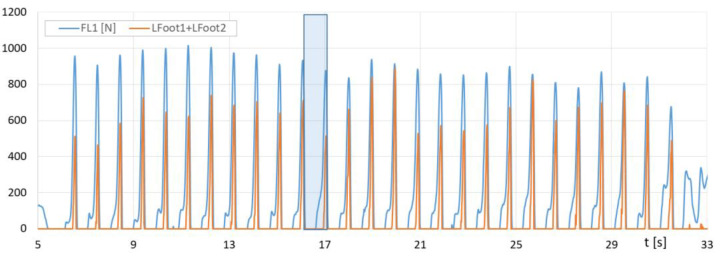
Fluctuations in forces *F_L1_* (F-Scan) and the sum of *Lfoot1 + Lfoot2* (FootM) metatarsal pressure with toes of walker’s 1 left foot on the ground during the selected pass with NW poles.

**Figure 15:**
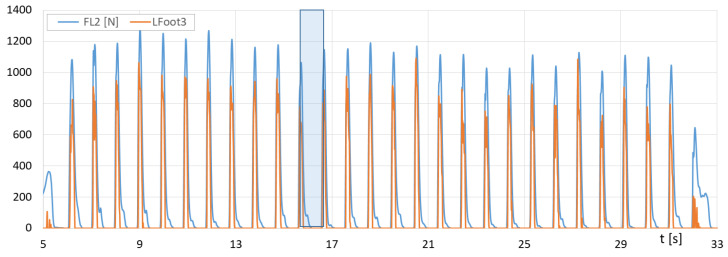
Fluctuations in forces *F_L2_* (F-Scan) and *Lfoot3* (FootM) heel pressure of walker’s 1 left foot on the ground during the selected pass with NW poles.

**Figure 16:**
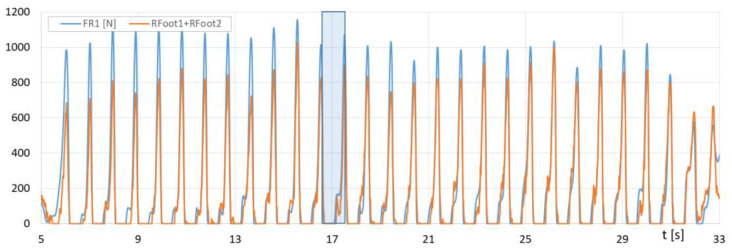
Fluctuations in forces *F_R1_* (F-Scan) and *Rfoot1 + Rfoot2* (FootM) of metatarsal pressure with toes of the right foot of walker 1 on the ground during the selected pass with NW poles.

**Figure 17:**
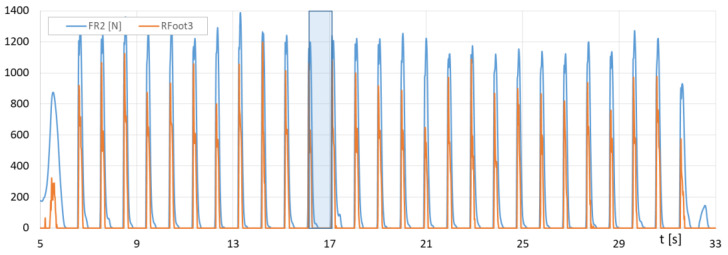
Fluctuations in forces *F_R2_* (F-Scan) and *Rfoot3* (FootM) of heel pressure of the right foot of walker 1 on the ground during the selected pass with NW poles.

**Figure 18:**
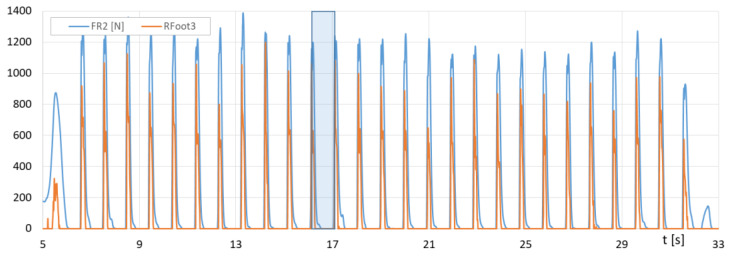
Fluctuations in the impact force of the left and right *Lforce*, *Rforce* poles against the ground during the selected pass of walker 1 with NW poles.

**Figure 19:**
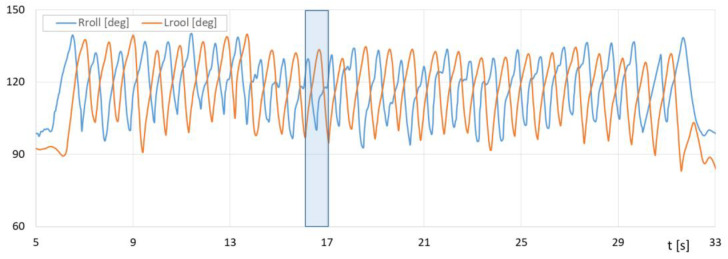
Fluctuations in the angle *Lroll*, *Rroll* of the left and right poles in the sagittal plane during the selected pass of walker 1 with NW poles.

**Figure 20:**
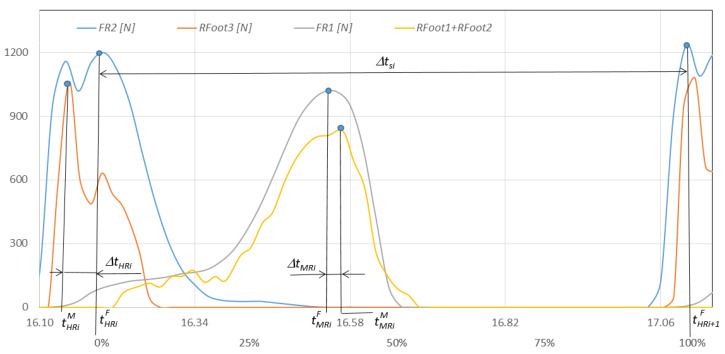
Fluctuations in forces *F_R1_*, *F_R2_* and *Rfoot1 + Rfoot2*, *Rfoot3* on the ground of the right foot during the selected gait cycle with NW poles for walker 1 in time t (in % of the gait cycle).

**Figure 21:**
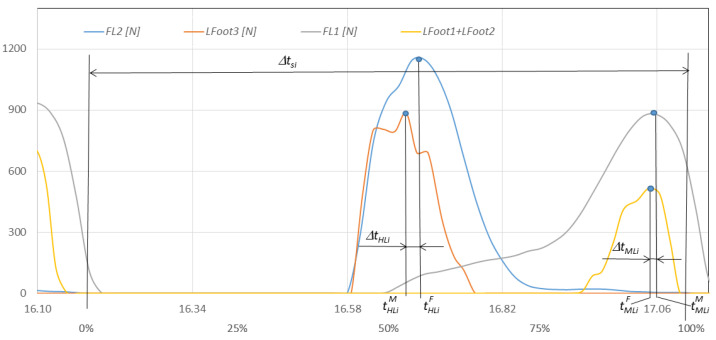
Fluctuations in forces *F_L1_*, *F_L2_* and *Lfoot1* + *Lfoot2*, *Lfoot3* on the ground of the left foot during the selected gait cycle with NW poles for walker 1 in time t (in % of the gait cycle).

**Figure 22:**
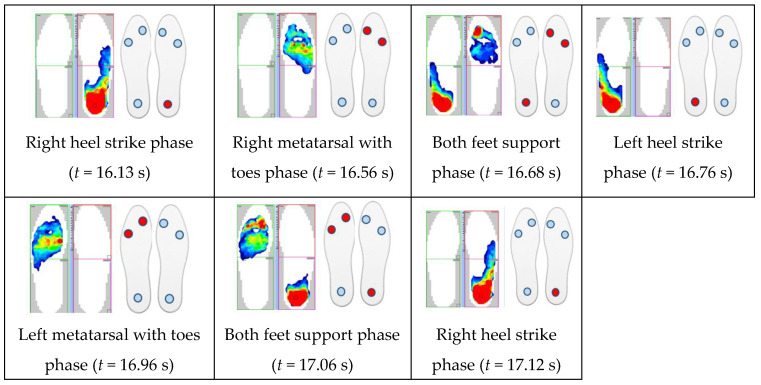
Graphical views of foot pressure maps illustrating the support and transfer phases of the selected gait cycle of walker 1 determined by using the F-Scan and FootM systems.

Experimental studies of NW gait measurements were carried out for a selected test group of walkers (Table 4) using the MNW system with PoleM and FootM modules and the additional F-Scan system. The obtained example characteristic results of the MNW suitability tests for the analysis of NW gait phases are shown in the following graphs for walker 1 (male 178 cm, 89 kg, Figure 14, Figure 15, Figure 16, Figure 17, Figure 18, Figure 19, Figure 20, Figure 21 and Figure 22). 

The graph In Figure 14 shows the force runs of *F_L1_* (measured with the F-Scan system) and the sum of *Lfoot1 + Lfoot2* forces (measured with the FootM system) of the metatarsal with the toes of the left foot on the ground, while Figure 15 shows the force runs of *F*_L2_ and *Lfoot3* (FootM system) of the heel pressure of the left foot of walker 1 on the ground during the selected pass with the NW poles.

Figure 16 presents the runs of *F_R1_* (F-Scan) and the sum of *Rfoot1 + Rfoot2* (FootM) forces of the right foot’s metatarsals with toes on the ground, while Figure 17 shows the runs of *F_R2_* (F-Scan) and *Rfoot3* (FootM) forces of the heel of the right foot on the ground during a selected NW pass.

Figure 18 shows the course of the recorded contact force of the poles on the ground as a function of time for a selected section of the NW march, and Figure 19 shows the course of changes in the angle of the poles’ inclination.

Figure 18 contains the runs of the impact forces of *Lforce* and *Rforce* of the poles against the ground measured with the PoleM module. Figure 19 provides a plot of the changes in *Lroll*, *Rroll* angles of the left and right poles measured with the PoleM module. 

With the results of gait measurements obtained with the NW mechatronic gait testing system, it was possible to perform analyses of the whole passage or identify individual gait cycles. By analyzing the selected passage of walker 1 with NW poles, the results of which are shown in Figure 14, Figure 15, Figure 16, Figure 17, Figure 18 and Figure 19, it was possible to distinguish 27 complete gait cycles in a time of 27.2 s. In order to present a detailed analysis of the gait with NW poles, an example of the 12th gait cycle was selected (blue box in Figure 14, Figure 15, Figure 16, Figure 17, Figure 18 and Figure 19), starting with the heel of the right foot striking the ground at 16.13 s and ending with the same heel striking the ground at 17.12 s. The graphs in Figure 20 and Figure 21 show the changes in ground forces of the right and left feet measured with the F-Scan and FootM systems during the selected gait cycle. When analyzing this cycle, it is possible to distinguish support phases: on the left foot, on two feet, on the right foot; and transfer phases: left and right lower limb. The different phases of the cycle are illustrated in Figure 22 by showing graphical maps of feet-on-ground contact forces obtained with the F-Scan system and highlighting the active pressure sensors obtained with the FootM system.

From the analysis of the waveforms of the forces of foot pressures on the ground of walker 1 (Figure 14, Figure 15, Figure 16, Figure 17, Figure 20 and Figure 21), it is clear that the developed MNW system and its FootM module measure the values of these pressures with some error compared to the measurements with the F-Scan system.

Based on Formulas (1)–(6), statistical analyses were performed by determining the values of basic NW gait parameters: mean errors $∆t_{s}$, $∆t_{HR}$, $∆t_{HL}$, $∆t_{MR}$, and $∆t_{ML}$ and their standard deviations $\sigma t_{s}$, $\sigma t_{HR}$, $\sigma t_{HL}$, $\sigma t_{MR}$, and $\sigma t_{ML}$. Calculations were performed for *n = 240* selected gait cycles from 10 passes of 10 walkers (Table 4). The results of the calculations are shown in Table 5.

All the walkers whose gait parameters are shown in Table 5 moved with an NW gait over the same 30 m distance. The mean gait cycle time $∆t_{s}$ was 0.95 +/−0.02.

The results showed that moments of heel-to-ground pressure strikes, determined with the developed FootM system. Relative to the F-Scan system, it had an error $∆t_{HR}$ of less than 6.24% for the right foot and an error of $∆t_{HL}$ < 5.96% for the left foot. The moments of impact of the metatarsals with the toes against the ground were determined with the FootM system, with an error of $∆t_{MR}$ < 0.73% for the right foot and $∆t_{ML}$ < 0.47% for the left foot.

The accuracy of determining metatarsal-to-ground impact moments ($∆t_{MR},∆t_{ML}$) with the FootM system was greater than the accuracy of determining heel-to-ground impact moments ($∆t_{HR},∆t_{HL}$). This was due to the design of the FootM system shoe insole (Figure 5), in which two force sensors are placed in the metatarsal area with the toes, while there is only one sensor in the heel area. The slight differences in the errors for the two feet measured with the right and left FootM system insoles were due to the individual placement of the insoles in the left and right shoes, along with non-symmetrical movements of the walker’s limbs, which translate into differences in the calculation of foot pressure fields.

The results of the measurements and the presented results of the statistical analyses allow us to conclude that the developed FootM foot pressure measurement system, based on three sensors per insole, allows the determination of bare heel and metatarsal pressures with toes against the ground. Furthermore, it does so with sufficient accuracy compared to the F-Scan system, in which each insole has 964 pressure sensors.

The heel and metatarsal toe forces against the ground measured with the FootM system allow the phases of the gait cycle (the phases of the leg support and transfer) to be determined correctly. They can be used effectively in the MNW system of mechatronic NW poles. Further analysis of NW gait phases was carried out only with the developed mechatronic MNW system and its PoleM and FootM modules.

The values of forces obtained with the MNW system, shown in the graphs, differed slightly quantitatively from those measured with the F-Scan system. The measurements of the MNW system should be interpreted as the change in foot pressure on the ground and not as the absolute contact force of the foot on the ground. Considering the use case of the proposed MNW system, it is absolutely sufficient to use the relative change in the value of the contact force of the foot on the ground.

In order to comprehensively analyze the NW gait cycle, it was necessary to take into account the patterns of movement of the NW poles in the sagittal plane of the walker and the patterns of impact forces of the poles against the ground, forces of heel pressure, and forces of metatarsal pressure with toes against the ground. 

**Figure 23:**
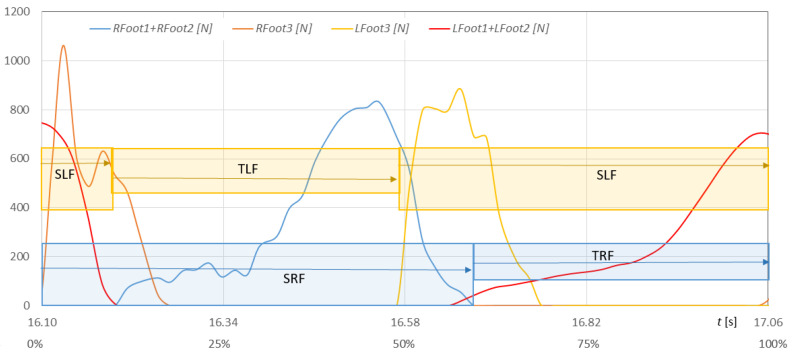
Fluctuations in forces *Rfoot1 + Rfoot2* and *Rfoot3* of the metatarsal and heel of the right foot and in forces *Lfoot1*+ *Lfoot2* and *Lfoot3* of the metatarsal and heel of the left foot on the ground during the selected gait cycle with NW poles for walker 1 in time t (in % of the gait cycle) (SLF—support phases of left foot, SRF—support phases of right foot, TLF—transfer phases of left foot, TRF—transfer phases of right foot).

**Figure 24:**
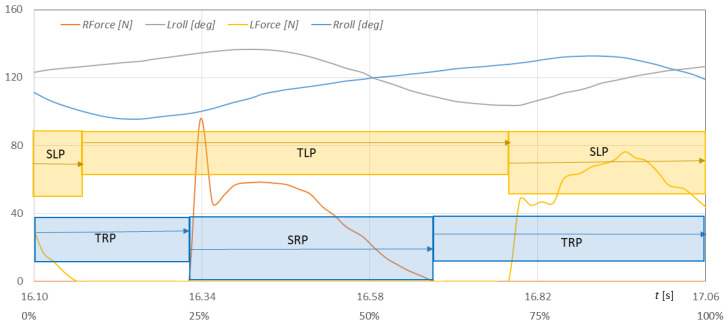
Fluctuations in the *Lroll*, *Rroll* angles of the pole inclination in the sagittal plane and the *Lforce*, *Rforce* forces of pole strokes at time *t* (in % of the gait cycle) during the selected gait cycle with the NW poles for walker 1 (SLP—support phases of left NW pole, SRP—support phases of right NW pole, TLP—transfer phases of left NW pole, TRP—transfer phases of right NW pole).

Figure 23 shows the waveforms of the heel and metatarsal with toes areas of both feet on the ground, and Figure 24 shows the waveforms of NW pole rotation angles and pole impact forces on the ground during the selected gait cycle for walker 1.

The runs were measured with the developed MNW system. The phases of support and transfer of the right foot could be determined from the force waveforms of the right heel (*Rfoot3*) and metatarsals with toes (*Rfoot1* + *Rfoot2*). The runs of Lfoot3 force and the sum of *Lfoot1* + *Lfoot2* forces determined the support and transfer phases of the left foot. The phases of movement of both feet in Figure 23 are marked with corresponding rectangles with descriptions (blue—phases of the right foot, yellow—phases of the left foot). The support phase on both feet can be quickly identified as the common part of the support phases on the right and left feet (Figure 23). For the selected cycle, the support phase to transfer phase of the right foot is divided 36% to 64%. The left foot’s phases are divided 37% to 63% concerning the length of the entire gait cycle.

The *Lforce*, *Rforce* force runs of the poles striking the ground allow us to identify the support (*Lforce*, *Rforce* > 0) and transfer (*Lforce*, *Rforce* = 0) phases of the right and left NW poles (blue rectangles—right pole phases, yellow rectangles—left pole phases). The NW pole support and transfer phases determined in Figure 24 make it possible to unambiguously determine the angular position of the cue at the moment of impact with the ground and to determine the ranges of changes in the rotation angles of the *Rroll* and *Lroll* cues during the execution of the NW gait phases. The support phase relative to transfer phase of the right NW pole is divided 36% to 64%. The phases of left NW pole are divided 30% to 70% concerning the length of the entire gait cycle.

In the graphs presented, it is possible to identify the individual phases of stride during the NW gait, the ranges of changes in the angles of the left and right poles, the forces and moments of the poles striking the ground, and the phases of support and transfer of the poles. The gait phases determined during measurements with the MNW system can be used to analyze the NW gait technique, specified and described in Section 2.

In the case of walker 1, for the individual cycles, a significant irregularity in the course of the NW pole contact forces is apparent, and the maximum values vary significantly. This indicates a lack of proper coordination of the movements performed as well as a lack of experience of walkers in the NW march. They definitely use too much force in the pole support phase of NW. This element clearly shows the advantage of using the proposed NW gait analysis system over the traditional training approach. While the coach observing the walker can easily notice inappropriate movements of the NW poles, it is practically impossible for the coach to objectively determine the fact that the contact pressure of the NW poles on the ground is too strong or weak. From the point of view of system design, it should be noted that the force sensors placed in the poles are characterized (PoleM module) by sufficient sensitivity and an adequate measurement range. 

The values of the maximum pole forces (106N) obtained from the measurements for walker 1 are comparable to the results of the NW male gait (116N) presented in the paper [19]. The force runs, divided into transfer and support phases, have qualitatively consistent characteristics.

When interpreting the NW pole inclination angles (Figure 19 and Figure 23), one can observe very similar ranges of changes in pole inclination (98–139°). Relating these values to the gait cycle, the angles of the poles in the moment of striking have far too small values. Walker 1 sets the NW pole almost vertically, which is incorrect. The pole movement of walker 1 is performed alternately with respect to the limb movement and is characterized by a slight asymmetry of the left and right sides. The ranges of changes in inclination angles considered in the individual cycles are characterized by a rather high variability. This is due to the lack of experience in NW gait and the lack of trained (established) repetition of the movements performed.

The selected results of the example gait cycles for walker 1, which are shown in the graphs above (Figure 20, Figure 21, Figure 23 and Figure 24), allow unambiguous identification of the gait phases (distinguishing the support and transfer phases of the foot) and the support and transfer phases of PoleM. This confirms the performance validity of the FootM sensory insoles and the PoleM pole module for different dynamic loading conditions. The proposed FootM solution is sufficient to separate the individual gait phases and provides the same accuracy in identifying the walking cycles as the commercial Tekscan F-Scan In Shoe measurement system.

## 4. Summary and Conclusions

This article has presented the development and capabilities of a new, unique mechatronic MNW system for NW gait analysis with respect to possible use in rehabilitation. Measurement of selected kinematic and dynamic factors of both the patient and the poles during the NW gait under natural conditions required the preparation of appropriate measurement tools. Accordingly, the developed mechatronic NW poles are equipped with a set of sensors monitoring selected kinematic and dynamic factors.

This article has presented the last stage of validation of the developed MNW system. It has been proven that the developed FootM module for measuring foot pressure on the ground can successfully determine the phases of NW gait cycles. Total NW measurements using the MNW system with the PoleM and FootM modules fully allow you to identify and monitor the movements of the walker’s limbs and poles. The measurement results of the walker’s gait can be used online/offline to check the correctness of the NW technique by a professional trainer. The proposed solution can be applied to poles made of any material (e.g., aluminum alloys, carbon). The additional elements installed on the pole do not require interference with the structure of the pole. After their removal, the NW pole remains in its original state. It is a great advantage because, after the end of the training period, the walker can use the same poles (without the measuring system).

Validation of the system confirmed its correct operation and its applicability in the widespread rehabilitation of medical conditions using NW marching. The results obtained confirmed that individual gait phases can be effectively identified, and the necessary kinematic and dynamic parameters of the NW gait can be determined. The developed measurement system allows rehabilitators and physiotherapists to quantitatively assess the quality of training, identify abnormalities in the implemented rehabilitation process, and introduce changes in the NW gait technique.

When analyzing gait where traditional poles have been changed to mechatronic ones, it is essential to consider whether the additional components added (shoe inserts, external wiring, backpack with measurement module, sensors and controllers attached to the standard pole) interfere with and cause unconscious changes in NW gait technique. These aspects of the impact of the system were investigated and analyzed by the authors. The results are presented in papers [27,28,29], demonstrating the lack of negative impact of the developed MNW system on NW gait patterns.

The MNW system allows the application of biological feedback in the process of developing the correct NW gait technique. The system provides the ability to output the measured signals and send them to a mobile device (smartphone/tablet). Equipping the mobile device with an application analyzing NW gait parameters will allow for online evaluation of the correctness of the gait technique, with feedback then sent to the walker. Such feedback in the form of sound/vibration will enable real-time correction of inaccurate limb and pole movements during the NW march. The authors are in the final stages of developing such an application enabling biological feedback (biofeedback).

The authors are convinced that the effectiveness, accessibility, low cost, possibility of independent exercise, and selection of exercise intensity according to individual capabilities are factors that recommend walking with mechatronic NW poles in the primary and secondary prevention of many diseases.

## Figures and Tables

**Table 1:** UNCDW-500N—the main technical parameters [25].

Rated Capacity	Non-Linearity	Weight	Hight	Diameter	Repeatability
500 N	1.0% R.O.	1.5 g	5 mm	7 mm	1.0% R.O.

**Table 2:** Essential technical specifications of FlexiForce A201 sensor [26].

Force Range	Linearity (Error)	Sensing Area	Thickness	Repeatability
0–445 N	<±3% of full scale	9.53 mm	0.203 mm	<±2.5%

**Table 3:** The main technical parameters of the Sensor 3000E insole [26].

Pressure Range (kPa)	Total No. of Sensels	Sensel Resolution (sensel/cm^2^)	Thickness (mm)	Row Spacing (mm)	Column Spacing (mm)
483–862	954	3.9	0.178	5.1	5.1

**Table 4:** Data of participants partaking in the study.

Number of Participants	Men	Female	Age	Body Weight (BW) (kg)	Body Height (cm)
10	6	4	43.9 ± 12.2	74.9 ± 11.7	171.4 ± 8.9

**Table 5:** NW gait parameter error values.

Parameter	Unit	Value
Δ*t_s_*	s	0.95
σ*t_s_*	s	+/−0.02
Δ*t_HR_*	%	6.24
σ*t_HR_*	%	+/−2.12
Δ*t_HL_*	%	0.73
σ*t_HL_*	%	+/−1.51
Δ*t_MR_*	%	5.96
σ*t_MR_*	%	+/−2.76
Δ*t_ML_*	%	0.47
σ*t_HL_*	%	+/−1.51

## Data Availability

The data used to support the findings of this study are available from the corresponding author upon request.
